# Protocol for assessing endolysosomal chloride channel function in mammalian cells using endolysosomal patch-clamp technique

**DOI:** 10.1016/j.xpro.2026.104826

**Published:** 2026-09-08

**Authors:** Yayu Wang, Lily Yeh Jan

**Affiliations:** 1 Department of Physiology, University of California, San Francisco, San Francisco, CA 94158, USA

**Keywords:** Biophysics, Cell Biology, Molecular Biology

## Abstract

Here, we present a protocol for assessing endolysosomal chloride channel function in mammalian cells using the endolysosomal patch-clamp technique. We describe steps for vesicle enlargement, fabrication and fire-polishing of high-resistance pipettes, and mechanical dissection and isolation of enlarged vesicles. We then detail procedures for giga-seal formation and configuration to whole-endolysosome modes.

For complete details on the use and execution of this protocol, please refer to Wang et al.^1^

## Highlights

• Preparation and isolation of enlarged endolysosomes for patch-clamp recordings
• Fabrication of high-resistance pipettes for endolysosomal electrophysiology
• Whole-endolysosome patch-clamp in multiple recording configurations
• Measurement of endolysosomal chloride channel function in mammalian cells

## Abstract (editor highlights)

Publisher’s note: Undertaking any experimental protocol requires adherence to local institutional guidelines for laboratory safety and ethics.

## Graphical abstract

**Figure:**
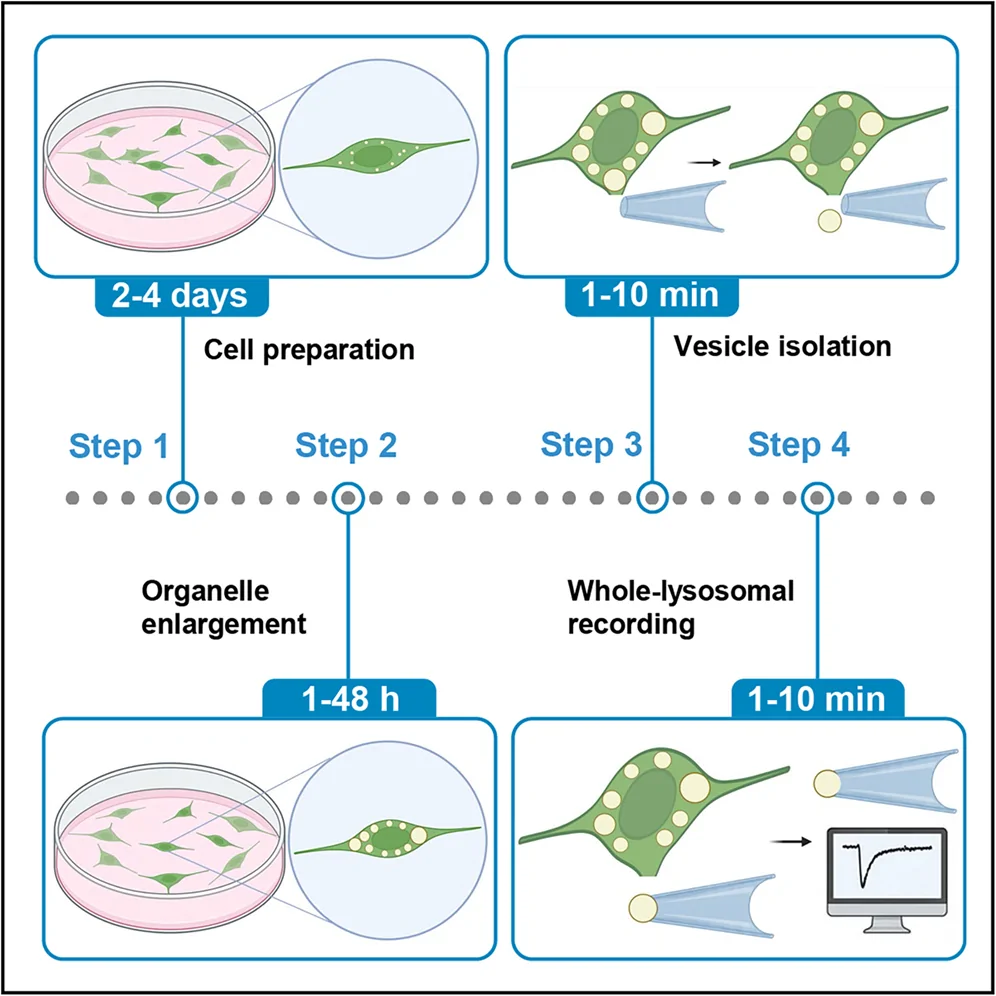

## Before you begin

The following protocol describes the steps required to record endolysosomal chloride currents and to assess lysosomal ion homeostasis in cultured mammalian cells following genetic manipulation of the CLN3 lysosomal chloride channel.^2^^,^^3^^,^^4^ In this protocol, endolysosome refers to the enlarged organelles generated by pharmacological or genetic manipulation, which comprise a heterogeneous population of late endosomes and lysosomes. Because these organelles cannot be reliably distinguished during whole-endolysosomal patch-clamp recordings, the term “endolysosome” is used throughout this protocol. This protocol is optimized for HEK293T cells and is designed to quantify proton-sensitive Cl^−^ conductance via endolysosomal patch-clamp recording. Nonetheless, the ability to combine high-resolution electrophysiology with live-cell imaging and genetic perturbations enables many additional applications.

This protocol can be used to investigate the impact of disease-associated CLN3 variants on lysosomal channel activity. When combined with complementary assays, such as ion-sensitive fluorescent probes and biochemical measurements, it can be used to examine changes in lysosomal chloride homeostasis and associated effects on lysosomal enzyme activity and protein degradation. Additionally, this approach has been used to study the regulation of lysosomal chloride levels by lysosomal membrane tension, vesicular trafficking, and autophagy-lysosome pathway dynamics under pharmacological or metabolic stress conditions.

### Innovation

This protocol presents an integrated workflow for directly measuring endolysosomal chloride conductance in intact mammalian cells.^5^^,^^6^^,^^7^ Traditional approaches rely primarily on fluorescence-based reporters or bulk biochemical measurements, which lack the temporal and electrophysiological resolution required to resolve channel-specific activity.^8^ Here, we combine endolysosomal patch-clamp electrophysiology with live-cell imaging and genetic perturbation to enable real-time, cell-specific quantification of proton-sensitive Cl^−^ currents.

In addition, customized ionic conditions allow precise control of luminal pH and chloride gradients, facilitating analysis of voltage- and proton-dependent channel gating. The integration of genetic manipulation (e.g., CLN3 variants) with patch-clamp recording further enables rapid structure-function studies and expands applications to lysosomal ion homeostasis and disease mechanisms.

### Preparation of coated coverslips

**Timing: 2 days (including incubation time)**

1. Sterilize microscope glass (12 mm) coverslips by autoclaving before use.
2. Immerse the coverslips in Poly-L-lysine coating solution and incubate for at least 24 h.
3. Remove the coating solution and wash the coverslips thoroughly with double-distilled water (ddH_2_O) five times.
4. Coated coverslips submerged in ddH_2_O can be stored at 22°C–25°C for up to one month.
5. Before cell seeding, transfer five coverslips into the 35 mm petri dish.
6. Allow the coverslips to air-dry completely in a laminar flow hood for at least 15 min prior to use.

**CRITICAL:** Coverslips should be fully dried before application of cells to promote proper attachment.

## Key resources table

**Table:** 

REAGENT or RESOURCE	SOURCE	IDENTIFIER
**Chemicals, peptides, and recombinant proteins**		

Sodium tetraborate decahydrate	Sigma Aldrich	Cat# S9640
Boric acid	Sigma Aldrich	Cat# B0394
Poly-L-lysine hydrobromide	Sigma Aldrich	Cat# P1399
Vacuolin-1	Sigma Aldrich	Cat# 673000
YM-201636	Cayman Chemical	Cat# 13576
Wortmannin	Cayman Chemical	Cat# 10010591
Latrunculin B	Cayman Chemical	Cat# 10010631
DMEM	Thermo Fisher Scientific	Cat# 10566016
Fetal Bovine Serum	Thermo Fisher Scientific	Cat# A5670701
Penicillin-Streptomycin	Thermo Fisher Scientific	Cat# 15140122
JetOPTIMUS DNA transfection reagent	Polyplus	Cat# 101000006
Hydrochloric acid (HCl)	Sigma Aldrich	Cat# 7647-01-0
Methanesulfonic acid (MSA)	Sigma Aldrich	Cat# 471356
N-methyl-D-glucamine (NMDG)	Sigma Aldrich	Cat# M2004
HEPES	Sigma Aldrich	Cat# H3375
MES hydrate	Sigma Aldrich	Cat# M8250
KCl	Sigma Aldrich	Cat# P9541
NaCl	Sigma Aldrich	Cat# S9888
KOH	Sigma Aldrich	Cat# 221473
Mg(OH)_2_	Sigma Aldrich	Cat# 63081
MgCl_2_·6H_2_O	Sigma Aldrich	Cat# M2670
CaCl_2_·2H_2_O	Sigma Aldrich	Cat# C5080
Glucose	Sigma Aldrich	Cat# G7021
K-gluconate	Sigma Aldrich	Cat# P1847
Na-gluconate	Sigma Aldrich	Cat# S2054
EGTA	Sigma Aldrich	Cat# E3889
TrypLE™ express enzyme (1×), no phenol red	Thermo Fisher Scientific	Cat# 12604039

**Experimental models: Cell lines**		

HEK293T	ATCC	Cat# CRL-3216; RRID:CVCL_0063

**Recombinant DNA**		

pcDNA3.1-CLN3-GFP	Thomas Braulke	Addgene Cat# 78110
pmCherry-C3-Rab5	This paper	N/A
pEGFP-N1-Lamp2	This paper	N/A
pSin-EF2-Rab7-puro	This paper	N/A
Fiji/ImageJ	National Institutes of Health	https://imagej.net/Fiji
Excel	Microsoft Office	https://www.microsoft.com/en-us/
Clampfit	Molecular Devices	https://www.moleculardevices.com/
OriginPro 2018	Originlab	https://www.originlab.com/

**Other**		

Patch clamp amplifier: Multiclamp 700B	Molecular Devices	N/A
Digitizer: Digidata 1550B	Molecular Devices	N/A
Multi-micromanipulator systems: MPC-200-ROE	Sutter Instrument	N/A
Micropipette puller: Sutter P-1000	Sutter Instrument	N/A
Glass: borosilicate capillaries	Sutter Instrument	Cat# BF150-75-10
Inverted microscope: Olympus I×73	Olympus	N/A
Osmometer: Osmotech	Advanced Instrument	N/A
Microforge: Narishige	Sutter Instrument	N/A

## Materials and equipment

**Table:** Vacuolin-1 stock solution (1 mM)

Reagent	Final concentration	Amount
Vacuolin-1	1 mM	1 mg
DMSO	N/A	1.73 mL
Total	N/A	1.73 mL

**CRITICAL:** Prepare Vacuolin-1 stock solution in an appropriate solvent according to the manufacturer’s solubility information (e.g., DMSO). Aliquot the solution after preparation to prevent product inactivation caused by repeated freeze-thaw cycles.

**CRITICAL:** Vacuolin-1 stock solutions can be stored at −80°C for up to 6 months or at −20°C for up to 1 month (stored under nitrogen).

**Table:** YM201636 stock solution (1 mM)

Reagent	Final concentration	Amount
YM201636	1 mM	1 mg
DMSO	N/A	2.139 mL
Total	N/A	2.139 mL

**CRITICAL:** YM201636 stock solutions can be stored at −80°C for up to 2 years or at −20°C for up to 1 year. Avoid moisture exposure and ensure the solution is fully dissolved before use.

**Table:** Wortmannin stock solution (1 mM)

Reagent	Final concentration	Amount
Wortmannin	1 mM	1 mg
DMSO	N/A	2.3343 mL
Total	N/A	2.3343 mL

**CRITICAL:** Wortmannin is unstable in aqueous solution and sensitive to moisture, light, and temperature. Stock solutions should be prepared in anhydrous DMSO, aliquoted to avoid repeated freeze-thaw cycles, and stored at −80°C for up to 1 year or −20°C for up to 1 month. Handle under minimal light exposure and limit air contact. Ensure complete dissolution before use, and prepare fresh working solutions prior to each experiment.

**Table:** Latrunculin B stock solution (1 mM)

Reagent	Final concentration	Amount
Latrunculin B	1 mM	0.5 mg
DMSO	N/A	1.2642 mL
Total	N/A	1.2642 mL

**CRITICAL:** Latrunculin B stock solutions can be stored at −80°C for up to 6 months or at −20°C for up to 1 month. Avoid moisture exposure and ensure the solution is fully dissolved before use.

**Table:** Poly-L-lysine coating solution (0.1 mg/mL)

Reagent	Final concentration	Amount
Poly-L-lysine	0.1 mg/mL	1 mg
Boric acid	80 mM	included in buffer
Borate	10 mM	included in buffer
ddH_2_O	N/A	to 10 mL
Total	N/A	10 mL

**CRITICAL:** The PLL stock solutions can be stored at −80°C for up to 6 months or at −20°C for up to 1 month. The pH of the borate buffer should be carefully adjusted (typically 8.5), as it can significantly affect the efficiency of Poly-L-lysine coating.

**CRITICAL:** The PLL solution is sterilized by filtration (0.22 μm).

### Pipette solution 1

Pipette solution is used as the intracellular recording solution for electrophysiological experiments. The concentrations listed below are the final concentrations after pH adjustment.

**Table:** 

Reagent	Final concentration	Amount
HCl	150 mM	625 μL
NMDG	150 mM	1.46 g
HEPES	10 mM	0.119 g
MES	10 mM	0.0976 g
ddH_2_O	N/A	to 50 mL
Total	N/A	50 mL

***Note:*** Prepare the solution in advance and store at −20°C for up to 1 year. Before use, allow the solution to reach 22°C–25°C. Adjust the pH to 4.6 by NMDG and osmolarity to 300 mOsm.

**CRITICAL:** Concentrated hydrochloric acid is corrosive. Handle with appropriate personal protective equipment and prepare the solution in a chemical fume hood. Add acid slowly to water while mixing.

**CRITICAL:** The pipette solution is sterilized by filtration (0.22 μm).

### Pipette solution 2

This solution is used as the luminal recording solution for chloride substitution experiments to assess the effect of cytosolic chloride concentration on CLN3 activity (Figure 4F). The concentrations listed below are the final concentrations after pH adjustment.

**Table:** 

Reagent	Final concentration	Amount
NaCl	145 mM	0.424 g
KOH	5 mM	0.014 g
MgCl_2_·6H_2_O	1 mM	0.0102 g
CaCl_2_·2H_2_O	1.5 mM	0.011 g
Glucose	10 mM	0.0901 g
HEPES	10 mM	0.119 g
MES	10 mM	0.0976 g
ddH_2_O	N/A	to 50 mL
Total	N/A	50 mL

***Note:*** Prepare the solution in advance and store at −20°C for up to 1 year. Before use, allow the solution to reach 22°C–25°C. Adjust the pH to 4.6 with methanesulfonic acid and the osmolarity to 300 mOsm.

**CRITICAL:** The pipette solution is sterilized by filtration (0.22 μm).

### Bath solution 1

Bath solution is used as the extracellular recording solution during electrophysiological experiments (Figures 4A and 4B). The concentrations listed below are the final concentrations after pH adjustment.

**Table:** 

Reagent	Final concentration	Amount
Methanesulfonic acid	149 mM	4.84 mL
HCl	1 mM	41.7 μL
NMDG	150 mM	14.64 g
HEPES	20 mM	2.38 g
ddH_2_O	N/A	to 500 mL
Total	N/A	500 mL

***Note:*** Prepare the solution in advance and store at 4°C for up to 6 months. Before use, allow the solution to reach 22°C–25°C. Adjust the pH to 7.2 by NMDG and osmolarity to 300 mOsm.

***Note:*** Dissolve the reagents in 80% of the final volume of ddH_2_O, then bring the solution to the final volume with additional ddH_2_O.

### Bath solution 2

This solution is used to reduce the cytosolic chloride concentration for chloride selectivity and reversal potential measurements (Figures 4C–4E). The concentrations listed below are the final concentrations after pH adjustment.

**Table:** 

Reagent	Final concentration	Amount
Methanesulfonic acid	140 mM	4.55 mL
HCl	10 mM	417 μL
NMDG	150 mM	14.64 g
HEPES	20 mM	2.38 g
ddH_2_O	N/A	to 500 mL
Total	N/A	500 mL

***Note:*** Prepare the solution in advance and store at 4°C for up to 6 months. Before use, allow the solution to reach 22°C–25°C. Adjust the pH to 7.2 with NMDG and the osmolarity to 300 mOsm.

***Note:*** Dissolve the reagents in 80% of the final volume of ddH_2_O, then bring the solution to the final volume with additional ddH_2_O.

### Bath solution 3

This solution is used as the extracellular recording solution for whole-endolysosome patch-clamp recordings under symmetrical high-chloride conditions and for chloride selectivity/reversal potential measurements (Figure 4C). The concentrations listed below are the final concentrations after pH adjustment.

**Table:** 

Reagent	Final concentration	Amount
HCl	150 mM	6.25 mL
NMDG	150 mM	14.64 g
HEPES	20 mM	2.38 g
ddH_2_O	N/A	to 500 mL
Total	N/A	500 mL

***Note:*** Prepare the solution in advance and store at 4°C for up to 6 months. Before use, allow the solution to reach 22°C–25°C. Adjust the pH to 7.2 with NMDG and the osmolarity to 300 mOsm.

***Note:*** Dissolve the reagents in 80% of the final volume of ddH_2_O, then bring the solution to the final volume with additional ddH_2_O.

### Bath solution 4

This solution is used as the extracellular recording solution for chloride substitution experiments to assess the effect of cytosolic chloride concentration on CLN3 activity (Figure 4F). The concentrations listed below are the final concentrations after pH adjustment.

**Table:** 

Reagent	Final concentration	Amount
KCl	140 mM	5.22 g
NaCl	5 mM	0.146 g
MgCl_2_·6H_2_O	2 mM	0.203 g
CaCl_2_·2H_2_O	0.5 mM	0.0368 g
EGTA	1 mM	0.19 g
HEPES	10 mM	1.19 g
ddH_2_O	N/A	to 500 mL
Total	N/A	500 mL

***Note:*** Prepare the solution in advance and store at 4°C for up to 6 months. Before use, allow the solution to reach 22°C–25°C. Adjust the pH to 7.2 with KOH and the osmolarity to 300 mOsm.

***Note:*** Dissolve the reagents in 80% of the final volume of ddH_2_O, then bring the solution to the final volume with additional ddH_2_O.

### Bath solution 5

This solution is used to reduce the cytosolic chloride concentration during chloride substitution experiments. The desired cytosolic chloride concentration (25 mM Cl^−^) is obtained by partially replacing the high-chloride bath solution with this low-chloride solution while maintaining constant pH and osmolarity (Figure 4F). The concentrations listed below are the final concentrations after pH adjustment.

**Table:** 

Reagent	Final concentration	Amount
K-gluconate	140 mM	16.40 g
Na-gluconate	5 mM	0.545 g
Mg(OH)_2_	2 mM	0.058 g
CaCl_2_·2H_2_O	0.5 mM	0.0368 g
EGTA	1 mM	0.19 g
HEPES	10 mM	1.19 g
ddH_2_O	N/A	to 500 mL
Total	N/A	500 mL

***Note:*** Prepare the solution in advance and store at 4°C for up to 6 months. Before use, allow the solution to reach 22°C–25°C. Adjust the pH to 7.2 with KOH and the osmolarity to 300 mOsm.

***Note:*** Dissolve the reagents in 80% of the final volume of ddH_2_O, then bring the solution to the final volume with additional ddH_2_O.

**Table:** HEK293T medium

Reagent	Final concentration	Amount
DMEM high glucose (contained GluMAX)	N/A	445 mL
FBS	10% (v/v)	50 mL
Penicillin-streptomycin (100×)	1 ×	5 mL
Total	N/A	500 mL

**CRITICAL:** The complete medium should be stored at 4°C for up to 6 months and warmed to 37°C before use.

## Step-by-step method details

### Preparation of HEK293T cells and transfection for endolysosomal recordings

**Timing: 2–4 days**

1. Maintain cells.
  a. Keep HEK293T cells in supplemented DMEM at 37°C in a humidified incubator with 5% CO_2_.
  b. Passage cells every 2 to 3 days to maintain healthy growth.

***Note:*** HEK293T cells should be passaged before reaching 80%–90% confluency to maintain healthy growth.

2. Cell detachment.
  a. Remove culture medium from a T25 flask.
  b. Add 1.0 mL of TrypLE™ express enzyme. Incubate at 37°C, 5% CO_2_ for 2 min.
  c. Check detachment under a microscope. If cells remain attached, incubate for an additional 1 min.

***Note:*** Avoid over-digestion, as prolonged exposure to TrypLE™ may reduce cell viability.

3. Quench trypsin.
  a. Add 4.0 mL of supplemented DMEM to dilute trypsin.
  b. Pipette the suspension up and down gently to ensure a single-cell suspension.

***Note:*** Gentle pipetting minimizes cell damage and improves uniform seeding.

4. Cell seeding.
  a. Transfer 0.5 mL of cell suspension (8 × 10^5^ cells) to a new 35 mm petri dish with 1.5 mL of supplemented DMEM.
  b. Incubate at 37°C, 5% CO_2_ for 1 to 2 days until about 80% confluent.

***Note:*** Optimal confluency (70%–80%) is critical for achieving consistent transfection efficiency.

5. Transfection (1 to 2 days before patch-clamp).
  a. Transfect cells with plasmid DNA encoding CLN3 using polyplus following the manufacturer’s instructions.
    i. For a 35 mm petri dish, mix 2.0 μg plasmid DNA (EGFP-CLN3) with 200 μL buffer.
    ii. Add 4 μL Polyplus reagent, mix gently and incubate for 10 min at 22°C–25°C.
    iii. Add the transfection mix dropwise to cells in 2.0 mL complete DMEM.
      ***Note:*** Use a single transfection reagent consistently throughout the study to ensure reproducibility.
      **CRITICAL:** Fluorophore-tagged endolysosomal channel proteins enable visualization and identification of endolysosomes, facilitating whole-endolysosome patch-clamp recordings.
  b. Incubate cells at 37°C, 5% CO_2_ for 24 h.
  c. After 24 h post-transfection, detach and re-plate as Step 2 to 3 for vesicle enlargement and recordings. Verify transfection efficiency by EGFP fluorescence before proceeding.
    ***Note:*** Re-plating improves cell morphology and accessibility for patch-clamp recordings.

### Enlargement of endolysosomal vesicles

**Timing: 1–48 h (depends on reagent and cell type)**

**Figure 1:**
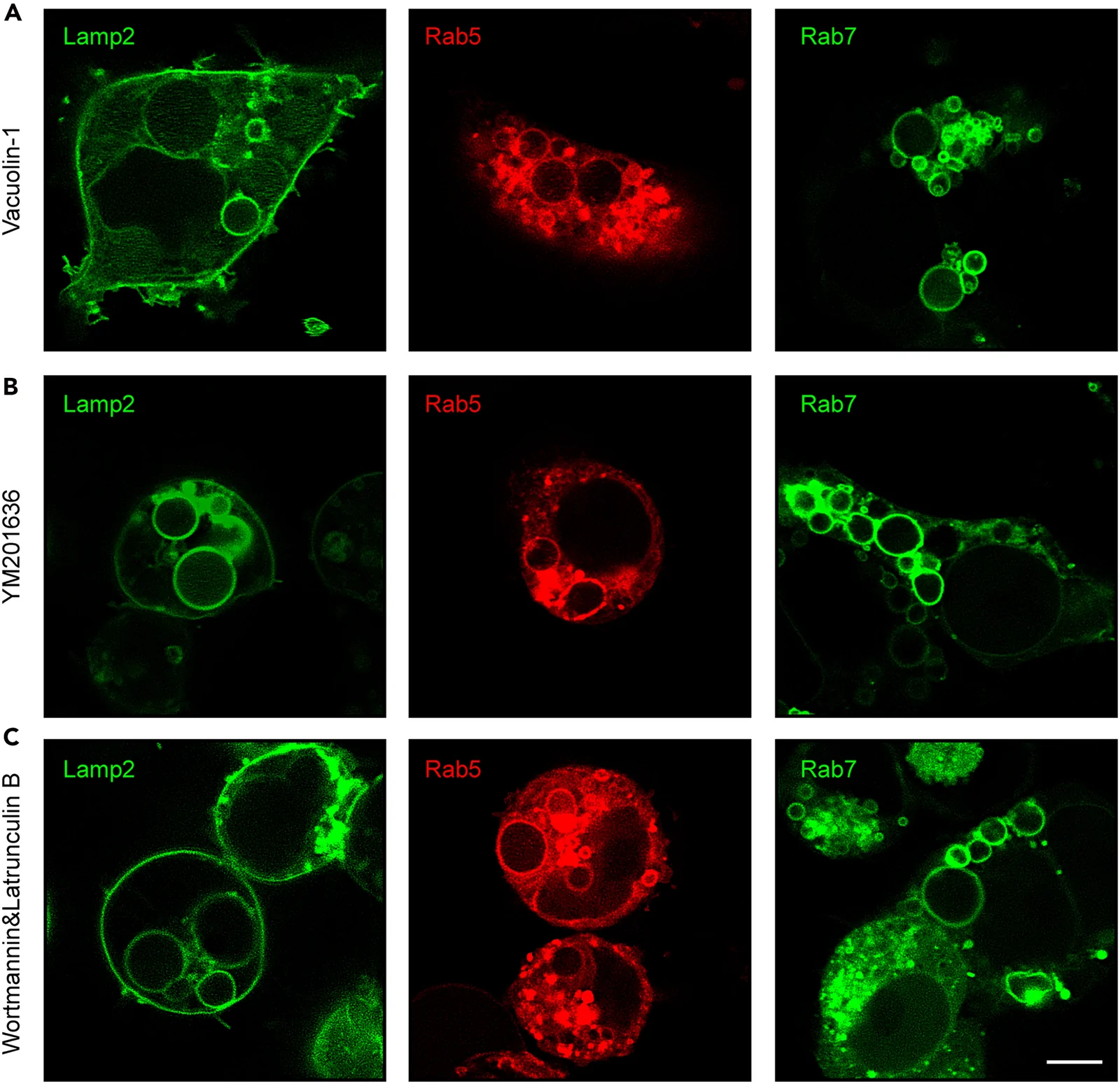
Pharmacological perturbation alters endolysosomal size Representative confocal images of cells stained for Lamp2 (green), Rab5 (red), and Rab7 (green) under the indicated treatments: (A) Vacuolin-1, (B) YM201636, and (C) Wortmannin in combination with Latrunculin B. Scale bar, 5μm.

This step induces enlargement of late endosomes and lysosomes to facilitate imaging, vesicle tracking, and functional analysis of endolysosomal compartments (Figure 1).

6. Treat cells with Vacuolin-1, YM-201636 or Wortmannin/Latrunculin B to induce endolysosomal enlargement.
  a. For Vacuolin-1 treatment^9^:
    i. Add Vacuolin-1 to a final concentration of 1 μM and incubate for 12–16 h.
    ii. Alternatively, use 1–5 μM for 1 h in certain cell types.
      ***Note:*** Vacuolin-1 induces relatively non-specific enlargement of endolysosomal compartments. The optimal concentration and incubation time may vary depending on the cell type.
  b. For PIKfyve inhibition using the YM-201636 compound^10^:
    i. In HEK293T cells, treat with 0.4 μM for 12–16 h or 0.8 μM for 2 h.
    ii. In macrophages, treat with 0.4 μM for 1–3 h.
      ***Note:*** YM-201636 specifically inhibits PIKfyve and preferentially enlarges late endosomes and lysosomes. Treatment conditions should be optimized to balance enlargement efficiency and cellular health.
      ***Note:*** Apilimod, another potent PIKfyve inhibitor, can also be used to induce enlargement of late endosomes and lysosomes. The choice between YM-201636 and apilimod may depend on the experimental design and cell type.
  c. For Wortmannin + Latrunculin B treatment:
    i. In HEK293 or COS-1 cells, treat with 200 nM Wortmannin + 10 nM Latrunculin B for 12–16 h.
    ii. Alternatively, use 100–500 nM Wortmannin + 1 to 2 μM Latrunculin B for 30–60 min in certain cell types.
7. Confirm vesicle enlargement by live-cell imaging.
  a. Use LAMP or Rab markers to visualize and verify enlargement of late endosomes/lysosomes.
  b. Optimize concentration and incubation time for each cell type.
  c. Compare different treatment conditions to rule out drug-induced artifacts.

***Note:*** The efficiency and extent of vesicle enlargement depend strongly on cell type, reagent concentration, and incubation time.

### Fabrication and fire-polishing of patch pipettes

**Timing: 1–10 min per pipette (pulling) + 1–3 min polishing**

The fabrication of glass pipettes is a critical step for successful endolysosomal patch-clamp recordings, as optimal pipette geometry and resistance are required for giga-seal formation on fragile intracellular organelle membranes.

8. Mount the glass capillary (e.g., BF150-75-10) onto the puller according to the manufacturer’s instructions.
9. Run a RAMP test to determine the appropriate HEAT value required to melt the glass under current experimental conditions.

**CRITICAL:** The HEAT parameter depends on filament condition, ambienttemperature (22°C–25°C), and glass type. Recalibration is required if the filament ages or experimental conditions change.

**Figure 2:**
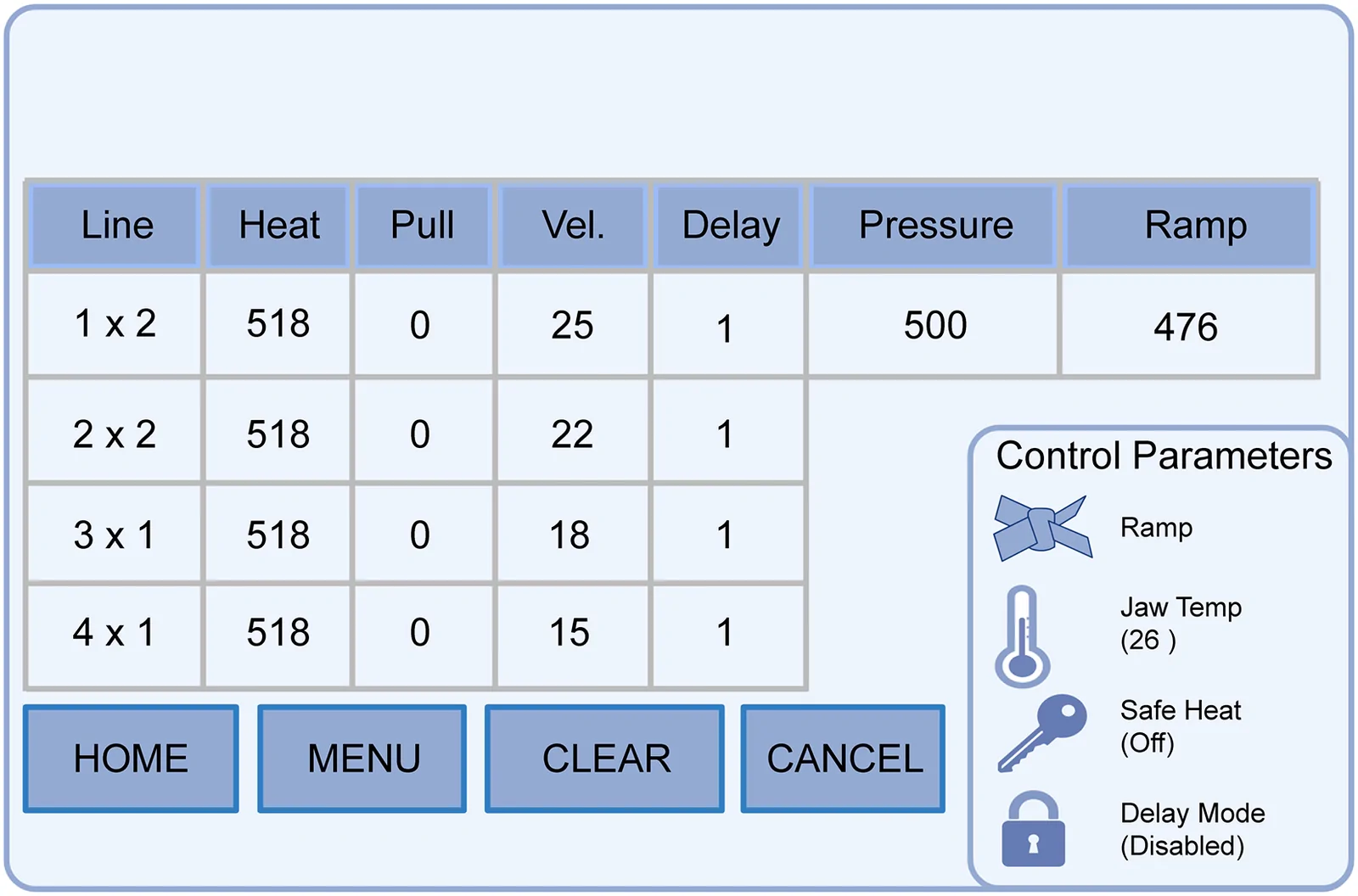
Patch-clamp recording parameters for lysosomal electrophysiology Representative configuration of acquisition parameters used for lysosomal patch-clamp recordings, including heat, pull, velocity (Vel.), delay, pressure, and ramp settings. Control parameters for ramp, jaw temperature, safe heat, and delay mode are shown on the right panel.

***Note:*** Pulling parameters must be optimized for each puller and glass type; values are not universally transferable.

***Note:*** We use a Sutter P-1000 puller with a six-cycle program, which provides high reproducibility for endolysosomal patch pipettes.

10. Program a six-cycle pulling protocol using optimized HEAT, velocity (VEL), and time (TIME) parameters in given Figure 2. Set HEAT based on Step 9.

11. Initiate the pulling program. Use six-cycle pulling program optimized for endolysosomal pipettes (aim for final pipette resistance 5–8 MΩ).

**CRITICAL:** Capillary separation should occur during the sixth cycle. Earlier separation typically produces overly sharp tips that are unsuitable for giga-seal formation.

12. Remove pipettes from the puller.
13. Inspect pipette tips under a microforge to assess tip diameter, shape, and symmetry. Discard obviously defective tips. Fire-polish remaining pipettes to obtain blunt tip geometry with inner path narrow and tip opening <0.9 μm; target final tip inner diameter <0.3 μm after polishing. Desired tip opening: 0.5–0.9 μm.

***Note:*** Pipette resistance (typically 5–8 MΩ) is primarily determined by tip geometry, glass properties, and internal solution composition.

**CRITICAL:** Proper pipette geometry is essential for achieving stable giga-seal formation on endolysosomal membranes. An excessively wide (>1 μm) or thick pipette tip can be corrected by increasing the HEAT parameter (sixth cycle) or the pulling velocity (VEL). In contrast, overly narrow or thin tips can be adjusted by reducing the HEAT or VEL parameters accordingly.

**CRITICAL:** Fine adjustment of HEAT and VEL (especially in the sixth cycle) is required for each pulling session.

14. Discard unsuitable pipettes (too sharp, too wide, too thin, asymmetric, or contaminated with debris).

**CRITICAL:** Approximately 50% of pipettes may fail quality control and should not be used for recordings.

***Note:*** Suboptimal pipettes may be repurposed for dissection or other applications.

15. Immediately fire-polish suitable pipettes using a microforge.

**CRITICAL:** Fire-polishing is essential to produce a smooth, slightly blunt tip, which improves seal formation and minimizes membrane damage.

**CRITICAL:** Use thick-walled glass; produce many pipettes (≥10–20) because 50% of those pipettes fail QC. Use polished pipettes within 6 h.

### Dissection and release of enlarged endolysosomes

**Timing: 1–10 min per vesicle**

16. Transfer coverslip with enlarged cells to the recording chamber and add 1 mL bath solution. Keep cells stable and perform dissections within 1 h of transfer.

**CRITICAL:** Perform dissections within 1 h after transfer; prolonged incubation leads to vesicle shrinkage, increased adhesion, and cell detachment.

17. Fill an isolation pipette with luminal solution and mount it on the headstage. Approach the cell at about 30° using the micromanipulator and select vesicles located near the cell edge and of appropriate size.

**CRITICAL:** Vesicles near the cell edge are easier to isolate; their unsuitable positioning significantly reduces success rate.

***Note:*** Vesicles should be <50% of the cell diameter; overly large vesicles are unstable, whereas smaller vesicles are difficult to patch. For whole-endolysosomal recordings, select enlarged endolysosomes with an apparent diameter of approximately 2–5 μm. Smaller vesicles are difficult to patch, whereas larger vesicles are more prone to rupture during isolation and recording.

18. Bring the pipette into contact with the plasma membrane near the target vesicle. Press gently and rapidly withdraw to create a membrane opening.

**CRITICAL:** Use rapid but controlled movement; slow or imprecise withdrawal reduces successful membrane incision.

19. Use the same pipette to push from the opposite side of the cell and squeeze the enlarged endolysosome out through the incision. Move the isolated vesicle away from the cell to avoid reattachment or contamination.

**CRITICAL:** Apply minimal and controlled force; excessive pressure may rupture the vesicle.

***Note:*** Isolated vesicles may transiently remain attached or drift back toward the cell.

20. Allow the vesicle to stabilize and confirm morphology (round shape, intact, thin and semi-transparent membrane) before proceeding to patch-clamp recording.

**CRITICAL:** Proceed only with intact vesicles; damaged membranes will not form stable giga-seals.

***Note:*** Vesicles should appear round with a thin, semi-transparent membrane.

### Giga-seal formation

**Timing: 1–3 min for giga-seal; break-in <1 min**

21. Use a freshly polished recording pipette and fill it with the appropriate luminal solution.

**CRITICAL:** Use freshly polished pipettes (<6 h); aged or contaminated tips significantly reduce seal success.

22. Mount the pipette onto the headstage of the amplifier.
23. Apply positive pressure (0.05–0.1 mL) using a 1 mL syringe and maintain constant pressure.

**CRITICAL:** Positive pressure prevents clogging and improves seal formation. Excessive pressure (>0.1 mL) may hinder subsequent break-in for whole-endolysosome configuration.

24. Set the micromanipulator MODE to 0. Lower the pipette into the bath and position it in the center of the visual field. Confirm visible outflow from the pipette tip.

***Note:*** Visible fluid flow indicates proper pressure and helps keep the tip clean before contacting the vesicle.

25. Apply repetitive test pulses (+5 mV, 50–100 ms) to monitor pipette resistance and seal formation.

***Note:*** A decrease in current amplitude indicates increasing seal resistance during giga-seal formation.

26. Set MODE to 6 and rapidly move the pipette to the top of the target vesicle (<30 s).

**CRITICAL:** Approach quickly but smoothly; prolonged exposure increases contamination risk and reduces seal probability.

27. Advance the pipette until vesicle movement is observed due to fluid flow.

***Note:*** Slight vesicle displacement indicates correct positioning and appropriate pressure.

28. Adjust the pipette offset potential to 0 mV.
29. Release the positive pressure immediately. The vesicle membrane should be drawn to the pipette tip and form a giga-seal (1–20 GΩ) within 1 s.

**CRITICAL:** Timing of pressure release is essential; delayed or gradual release reduces giga-seal formation efficiency.

**CRITICAL:** Only intact, stable vesicles will form high-resistance seals.

### Recording configurations after giga-seal formation

**Figure 3:**
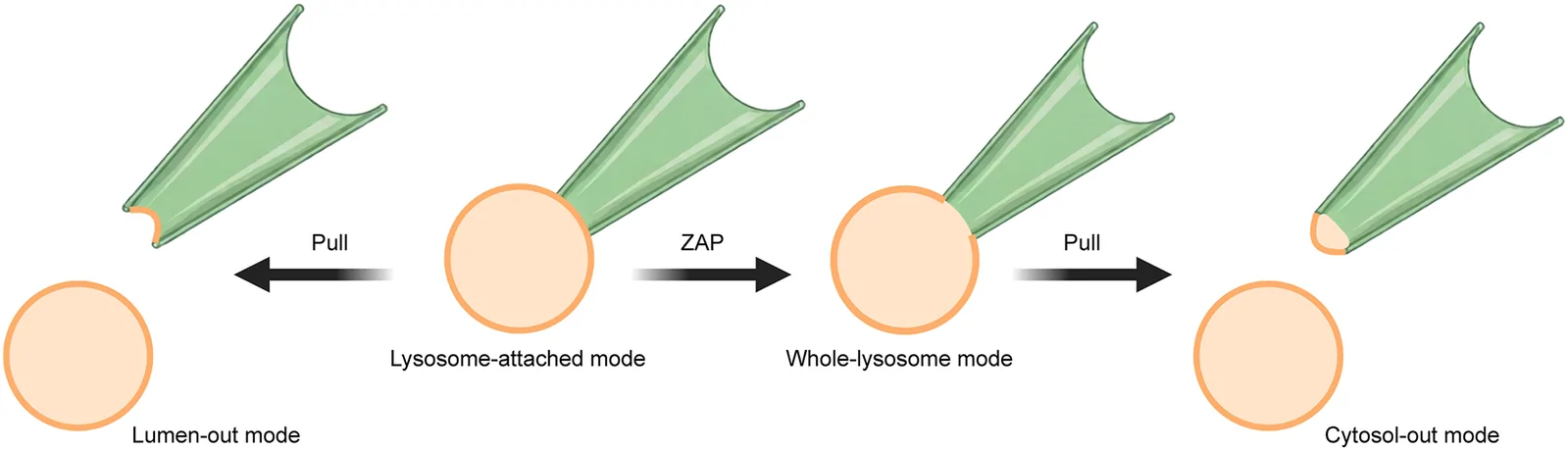
Single-lysosome recording configurations Single-lysosome recordings can be performed in four configurations: endolysosome-attached, whole-lysosome, lumen-out, and cytosol-out modes. The endolysosome-attached and whole-endolysosome modes represent intact organelle recording configurations, whereas lumen-out and cytosol-out modes are obtained after membrane excision, exposing either the luminal or cytosolic side of the lysosomal membrane to the bath solution.

**Timing: 1–5 min, depending on the selected recording configuration** (Figure 3)

After formation of a stable giga-seal, proceed with the desired recording configuration as outlined below.

30. Endolysosome-attached mode (single-channel recording)
  a. Maintain the giga-seal without membrane rupture and proceed directly to single-channel recording using step protocols.

***Note:*** In this configuration, the membrane potential of the endolysosome is unknown; therefore, the applied voltage does not reflect the absolute transmembrane potential.

**CRITICAL:** Current polarity is opposite to that observed in whole-endolysosome recordings.

31. Lumen-out configuration (excised patch)
  a. Quickly retract the pipette to excise the membrane patch from the vesicle.

**CRITICAL:** The excised patch may reseal into a vesicle; remove the pipette from solution for 1–5 s to rupture the outer membrane if necessary.

***Note:*** This configuration exposes the luminal side of the membrane to the bath solution, allowing direct control of luminal conditions.

32. Whole-endolysosome configuration.
  a. Apply a ZAP pulse using the amplifier default voltage setting with an initial duration of 25 μs to rupture the membrane patch and establish whole-endolysosome mode. If break-in is not achieved, gradually increase the pulse duration (e.g., to 50, 100, or 200 μs) as needed.

**CRITICAL:** Increase the ZAP pulse duration gradually before considering an increase in voltage to minimize damage to fragile endolysosomal vesicles. Stop increasing the pulse once successful break-in is achieved.

***Note:*** Successful break-in is indicated by the appearance of capacitive transients and a decrease in series resistance. If break-in cannot be achieved after repeated attempts, discard the vesicle and repeat the recording using a new endolysosome.

33. Cytosol-out configuration (excised patch from whole-endolysosome)
  a. After establishing whole-endolysosome mode, gently retract the pipette to excise a membrane patch from the vesicle.

**CRITICAL:** Retraction should be rapid and controlled to obtain a stable excised patch.

***Note:*** In this configuration, the cytosolic side of the membrane faces the bath solution, allowing direct control of cytosolic conditions.

### Recording protocols

**Timing: 1–5 min per recording protocol (the duration varies)**

After establishing the whole-endolysosome recording configuration, proceed with the corresponding recording protocol (**whole-endolysosome recordings**).

34. Compensate capacitance transients using amplifier circuitry until series resistance is <100 MΩ and capacitance is within 0.1–8 pF.

***Note:*** If capacitance compensation fails (e.g., no capacitance value is displayed or autocompensation error occurs), do not proceed with recording.

35. Select the recording mode (voltage-clamp recordings or current-clamp) and perform recordings as below.
  a. For voltage-clamp recordings:
    i. Set an appropriate holding potential depending on the channel and experimental conditions.
    ii. Apply voltage-step protocols using Clampex and record currents.
      ***Note:*** Recordings are typically performed at 22°C–25°C unless otherwise specified.
  b. For current-clamp recordings:
    i. Set I = 0 mode to disconnect external current input.
      **CRITICAL:** Ensure the holding current is set to 0 before switching modes; non-zero holding current may disrupt the giga-seal.
    ii. Load or design an appropriate current-clamp protocol.
    iii. Switch to current-clamp (IC) mode.
    iv. Monitor membrane potential and adjust tuning parameters to generate a stable sawtooth voltage response (∼10 mV amplitude).
    v. Enable automatic reduction of series resistance compensation if oscillations occur.
    vi. Adjust pipette capacitance neutralization until slight overshoot appears.
      ***Note:*** Overcompensation may induce oscillations and damage the vesicle; reduce slightly from the optimal value.
    vii. Perform series resistance compensation (auto or manual adjustment).
    viii. Acquire voltage recordings using the selected protocol.
      For cytosol-out recordings (optional), proceed as follows: After completing the whole-endolysosome recording configuration, slowly retract the pipette to obtain a stable excised membrane patch to establish cytosol-out configuration (**single-channel recordings**).
      **CRITICAL:** The membrane fragment should reseal at the pipette tip to form a patch with the cytosolic side exposed to the bath solution.
36. Set the headstage feedback resistor to 50 GΩ and enable headstage cooling to minimize noise.

**CRITICAL:** Turn on headstage cooling at least 1 h before recording to ensure signal stability.

37. Acquire single-channel currents using voltage-step protocols and verify channel activity.

***Note:*** If no channel activity is observed, discard the recording and repeat with a new vesicle.

## Expected outcomes

Typical current traces are observed as the patch pipette enters the recording solution and approaches the target vesicle. Properly fabricated pipettes used for endolysosomal patch-clamp recordings typically exhibit resistances of 5–8 MΩ. Following seal formation, the seal resistance increases to the giga ohm range (typically 1–16 GΩ), indicating successful giga-seal formation.

Upon establishing the whole-endolysosome configuration, capacitive transients become apparent, reflecting electrical access to the vesicle lumen. From these transients, series resistance and membrane capacitance can be estimated. Under typical conditions, series resistance ranges from 10 to 100 MΩ, and capacitance ranges from 0.1 to 8 pF.

**Figure 4:**
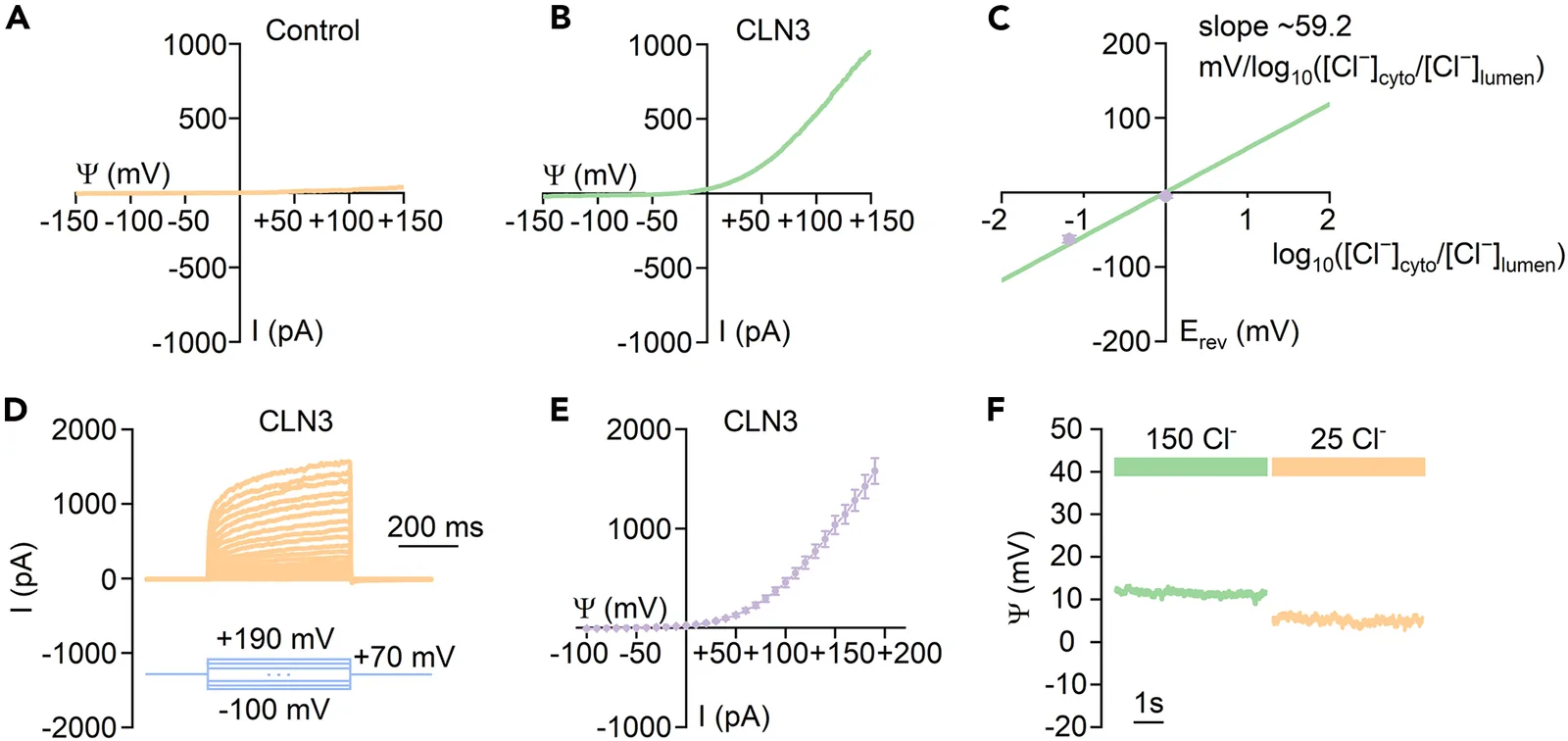
Representative whole lysosome recordings (A and B) In whole-endolysosome recordings, membrane potential is defined as the cytosolic potential relative to the luminal potential. Representative current-voltage (I-V) relationships recorded from non-transfected control cells (A) and CLN3-expressing (B) endolysosomes under asymmetric chloride conditions. The bath solution contained (in mM): 149 methanesulfonic acid (HMS), 20 HEPES, and 1 HCl, adjusted to pH 7.2 with NMDG. The pipette solution contained (in mM): 150 HCl, 10 HEPES, and 10 MES, adjusted to pH 4.6 with NMDG. CLN3-expressing endolysosomes exhibited pronounced outward rectification compared with non-transfected controls. (C) Reversal potentials measured under asymmetric (10 mM cytosolic Cl^−^/150 mM luminal Cl^−^) and symmetrical (150/150 mM Cl^−^) chloride conditions (purple dots). Data are presented as mean ± SEM. (D) Representative current traces recorded from CLN3 under step voltage protocols (−100 mV to +190 mV), illustrating time-dependent current activation. The bath solution contained (in mM): 140 methanesulfonic acid (HMS), 20 HEPES, and 10 HCl, adjusted to pH 7.2 with NMDG. The pipette solution contained (in mM): 150 HCl, 10 HEPES, and 10 MES, adjusted to pH 4.6 with NMDG. Scale bar, 200 ms. (E) Averaged I-V relationship of CLN3-mediated currents. Data are presented as mean ± SEM from 11 independent recordings (n = 11), confirming the voltage-dependent current profile. (F) Effect of cytosolic chloride concentration on CLN3 activity. Representative current traces recorded under symmetric conditions of 150 mM Cl^−^ (green) and reduced chloride (25 mM Cl^−^, orange), showing altered current amplitude. The bath solution contained (in mM): 140 KCl, 5 NaCl, 2 MgCl_2_, 0.5 CaCl_2_, 1 EGTA, and 10 HEPES (pH adjusted to 7.2 with KOH). The 25 mM Cl^−^ concentration was adjusted by partial substitution of the bath solution with an appropriate amount of low chloride solution containing (in mM): 140 K-gluconate, 5 Na-gluconate, 2 Mg(OH)_2_, 0.5 CaCl_2_, 1 EGTA, and 10 HEPES (pH adjusted to 7.2 with KOH). The pipette solution contained (in mM): 145 NaCl, 5 KOH, 1 MgCl_2_, 1.5 CaCl_2_, 10 glucose, 10 HEPES, and 10 MES (pH adjusted to 4.6 with methanesulfonic acid).

In whole-endolysosome recordings, membrane currents can be evoked using voltage-step or voltage-ramp protocols. These CLN3-mediated currents are typically in the picoampere (pA) range and are activated at positive voltages (Figures 4A–4E). Representative whole-endolysosome current traces and the corresponding current-voltage (I-V) relationship demonstrate pronounced outward rectification (Figures 4B, 4D, and 4E). Under asymmetric chloride conditions (10 mM cytosolic Cl^−^ and 150 mM luminal Cl^−^), the reversal potential was −62.8 ± 4.8 mV (n = 11), which is close to the theoretical chloride equilibrium potential (−69.5 mV) calculated using the Nernst equation at 25°C. Under symmetrical chloride conditions (150 mM Cl^−^ on both sides), the reversal potential shifted to −5.4 ± 2.3 mV (n = 5), consistent with the expected chloride equilibrium potential of approximately 0 mV (Figure 4C). These results confirm that the recorded currents are predominantly carried by chloride ions. High-quality recordings (achieved after a successful break-in at Step 29) must exhibit low baseline noise and minimal leak currents at hyperpolarized potentials. This confirms a stable Giga-seal and ensures the observed outward currents reflect true CLN3 activity.

In current-clamp mode, changes in membrane potential can be monitored in response to alterations in ionic compositions (Figure 4F). For instance, shifting the cytosolic-side chloride concentration from 150 mM to 25 mM induces a dynamic change in membrane potential. In excised or attached patch configurations, single-channel activity may be observed as discrete step-like current transitions between closed and open states. These events are characterized by defined amplitude levels and reproducible kinetics, provided that the signal-to-noise ratio is sufficiently high.

## Quantification and statistical analysis

Data analysis was performed using ImageJ, Clampfit (Molecular Devices), OriginPro (OriginLab) and Excel (Microsoft). Current traces were analyzed using Clampfit and OriginPro. Before seal formation, the pipette offset was adjusted to zero using the amplifier offset control. No additional offline baseline correction or leak subtraction was performed during data analysis. Only recordings with stable giga-seals, stable baseline currents, minimal leak currents, and stable series resistance throughout the recording were included in the analysis. Recordings exhibiting unstable baseline currents, excessive leak currents, loss of the giga-seal, or substantial changes in series resistance were excluded. Results are presented as mean ± SEM.

## Limitations

This protocol requires pharmacological or genetic enlargement of endolysosomes, which may alter membrane composition, lipid distribution, or regulatory protein interactions. Vacuolin-1 and YM201636 may affect trafficking pathways or phosphoinositide balance, potentially influencing channel behavior. Mechanical isolation introduces selection bias toward larger and more mechanically stable vesicles. Smaller or more fragile endolysosomes may be underrepresented in recordings. Whole-endolysosome configuration allows washout of luminal or cytosolic modulators, which may affect channel gating over time. Regulatory proteins or second messengers associated with lysosomal membranes may be diluted during recording. This protocol is technically demanding and requires extensive optimization of pipette fabrication and micromanipulation. Very small organelles or complex membrane systems such as the endoplasmic reticulum cannot be reliably accessed using this approach.

## Troubleshooting

### Problem 1

Enlarged lysosomes are not observed after Vacuolin-1, YM201636 or Wortmannin + Latrunculin B treatment (related to Step 6).

### Potential solution

Verify drug concentration and incubation time. Optimize concentration (between 0.5–5 μM) depending on cell type. Confirm cell viability and ensure cells are 40%–70% confluent at time of treatment. Test an alternative enlargement strategy (e.g., Rab5Q79L expression).

### Problem 2

Isolated vesicles rupture during mechanical dissection (related to Steps 16–20).

### Potential solution

Reduce mechanical force applied during extrusion. Select vesicles positioned near the cell periphery. Use a smoother and slightly blunter isolation pipette tip. Ensure cells are firmly attached to poly-L-lysine coated coverslips.

### Problem 3

Giga-seal cannot be established (related to Steps 21–29).

### Potential solution

Confirm that pipette tips are properly fire-polished and not excessively sharp. Reduce the positive pressure prior to contact with the cell. Ensure that the bath solution is clean and free of debris (e.g., by filtration). Replace the pipette if its resistance falls outside the range of 5–8 MΩ.

### Problem 4

Break-in fails after ZAP pulse (related to Step 29).

### Potential solution

Increase ZAP duration incrementally (50–100 μs). Avoid excessive positive pressure prior to break-in. Ensure vesicle diameter is ≥3 μm.

### Problem 5

Seal becomes unstable during recording (related to Steps 34 to 35).

### Potential solution

Move vesicle slightly upward after giga-seal formation to stabilize geometry. Minimize bath turbulence. Avoid excessive solution exchange during recording.

## Resource availability

### Lead contact

Further information and requests for resources and reagents should be directed to and will be fulfilled by the lead contact, Lily Yeh Jan (lily.jan@ucsf.edu).

### Technical contact

Technical questions on executing this protocol should be directed to and will be answered by the technical contact, Yayu Wang (yayu.wang@ucsf.edu).

### Materials availability

This study did not generate new unique reagents.

### Data and code availability

This study did not generate or analyze datasets/code.

## Acknowledgments

We thank all members of the Jan lab for valuable discussions. This work was supported by the 10.13039/100000002NIH grant to L.Y.J. (R35NS122110).

## Author contributions

Y.W. performed both the experiments and the analyses. L.Y.J. and Y.W. wrote and edited the manuscript.

## Declaration of interests

The authors declare no competing interests.
